# Effect of Nb/B addition on the flow behavior and mechanical properties of low-carbon steel using compact strip production

**DOI:** 10.1038/s41598-024-74927-y

**Published:** 2025-01-03

**Authors:** Ahmed Refaee, Eman El-Shenawy, Aly El Domiaty, Abdalla M. Abdalla, Reham Reda

**Affiliations:** 1 Department of Mechanical Engineering, Faculty of Engineering, Suez Canal University, Ismailia, 41522 Egypt; 2 Al Ezz Dekheila Steel Company (EZDK), El-Dekheila, Alexandria, 21537 Egypt; 3 Plastic Deformation Department, Metal Technology Institute, Central Metallurgical R& D Institute (CMRDI), Cairo, 11421 Egypt; 4 Department of Mechanical Engineering, Faculty of Engineering, Suez University, P.O.Box: 43221, Suez, Egypt

**Keywords:** Nb/B low-carbon steels, Compact strip production (CSP), Gleeble physical simulation, No-recrystallization temperature (T_nr_), Flow stress, Microstructure, Mechanical engineering, Metals and alloys

## Abstract

This work examines the effects of Nb and Nb-B additives on the high-temperature flow behavior and mechanical properties of low-carbon steel. The base, 0.015% Nb-bearing (15Nb alloy), and 0.015% Nb-30 ppm B-bearing (15Nb30B alloy) low-carbon steels were manufactured at Al Ezz Dekheila Steel Company using the compact strip production line (CSP). The mean flow stress log data from the CSP line was utilized to determine the no-recrystallization temperature. The microstructure and high-temperature flow behavior were studied using thermomechanical controlled processing at different deformation temperatures with a 40% reduction using the Gleeble physical simulator. The phase evolution and precipitation state at various temperatures were studied15Nb30B alloy reveals lower flow stress at high processing temperature in comparison with 15Nb alloy. This is attributed to the early precipitation of BN, which led to restricted Nb-based precipitates. The addition of B to Nb-bearing steel slightly refines the grain size of the as-rolled alloy, which in turn has a beneficial effect on its strength. The research results highlight the industrial benefit of adding boron to Nb-bearing low C-steel in terms of reducing the rolling load and reducing the finishing rolling temperature while maintaining the mechanical properties of the as-rolled strips.

## Introduction

The approach of combining the continuous casting and hot rolling of thin slabs is known as compact strip production technology (CSP). It is commonly used to produce hot strips directly from a continuous cast slab to reduce energy consumption and boost steel productivity. It has been used all over the world with industrialized CSP continuous casting and rolling manufacturing lines^1^.

The no-recrystallization temperature (T_nr_), which is the temperature at which recrystallization begins to be inhibited during hot processing, is the outcome of the interaction between hot deformation, recrystallization, and precipitation. Determining the T_nr_ is an essential step in designing controlled hot rolling regimes^2–5^, as it is the temperature below which austenite begins to accumulate strain (pancaking)^2,5^. When finishing rolling passes occur at temperatures lower than T_nr_, higher dislocations density pancaked austenite grains form. This structure can serve as a potential site for ferrite nucleation, resulting in the formation of a fine microstructure that enhances the mechanical properties when compared to the microstructure and properties resulting from processing at a temperature above T_nr_. The T_nr_ depends on composition, strain rate, effective strain, inter-pass time, and reheating temperature^2–4^. The value of T_nr_ drops as a result of the promotion of static recrystallization throughout the cooling process by increases in pass strain, strain rate, and interpass time. The T_nr_ is more impacted by the pass strain and interpass time than by the strain rate. T_nr_ increases with inter-pass duration until precipitation coarsening happens^2–4^. Higher T_nr_ values were obtained by increasing the reheating temperature; this effect is associated with higher amounts of austenite supersaturation before deformation^4^.

As Nb is the most effective alloying element that retards the recrystallization process by raising the non-recrystallization temperature (T_nr_), it has a significant impact on the thermo-mechanical controlled process (TMCP) of steel alloys^5–8^. Two different mechanisms were proposed to describe the behavior of Nb in the retardation of recrystallization during processing. The first mechanism is called the solute drag effect, with this mechanism, the solute Nb interacts with γ grain boundaries, reduces the mobility of grain boundaries, and hence retards the recrystallization kinetics^4,9^. This mechanism was observed in low-pass strains^4^. The second mechanism is the strain-induced precipitation pinning effect. In this mechanism, the Nb(C, N) precipitates during deformation and pins the grain boundaries, causing recrystallization retardation^4,5,9^. This mechanism was observed in high-pass strains^4^. Therefore, Nb is the most efficient grain refining element for low-carbon hot-rolled steels^7,10,11^.

Transverse cracking is one of the key issues influencing the steel continuous casting process since it degrades the quality of the finished product and, in extreme situations, can lead to break casting out resulting in cutting casting sequence, which has a negative impact on productivity, downtime, maintenance costs, and, in certain situations, operator safety. It is thought that the cracks originate in mold and that they subsequently propagate in the solid state. Mold oscillation is one of the crucial elements of continuous casting that prevents the slab surface from sticking. Cracks may begin at the oscillation points in some circumstances^12,13^. If the strand’s surface temperature has low ductility during the straightening process after it is released from the mold, transverse cracks are stimulated. Numerous investigations have shown that the addition of Nb causes deterioration of ductility and can result in transverse cracks because of Nb(C, N) precipitation^14,15^.

To increase hot ductility, B is added to Nb-bearing steel^16–18^. According to Hannerz^16^, B increases hot ductility by interacting with N in a manner similar to that of Ti. As a result, less N will be available for the harmful Nb(C, N) to precipitate. According to Kim et al.^17^, B increases hot ductility due to the precipitation of coarse Fe_23_(B, C)_6_, which serves as the preferred location for ferrite (α) intragranular nucleation. Because of the soft intragranular ferrite, this process decreases the number of voids and cavities created at grain borders, making the interior of the γ grain more flexible. In order to increase steel’s hardenability, B is also added. Hannula et al.^19^ have shown that B causes the ferrite and bainite transformation curves in the CCT diagram to move to the right, and numerous other studies have produced similar findings^20–22^.

This study aimed at investigating the industrial benefit of adding B to Nb-bearing low C-steel by analyzing its mechanical properties and processing costs in terms of the rolling load and temperature utilizing the CSP process. Using industrial data from EZDK- hot strip mill (HSM) logs, TMCP utilizing Gleeble physical simulator, thermodynamic calculations, scanning electron microscopy (SEM), and energy dispersive spectroscopy (EDS), this work examines the impact of Nb/B on the mechanical properties, high-temperature flow behavior, and microstructure of low carbon steel.

## Materials and methods

**Figure 1:**
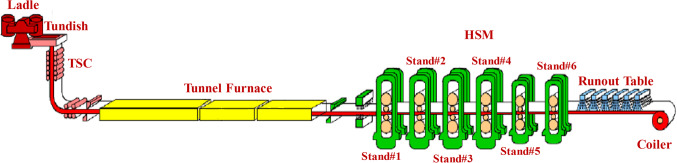
EZDK-CSP line scheme.

**Figure 2:**
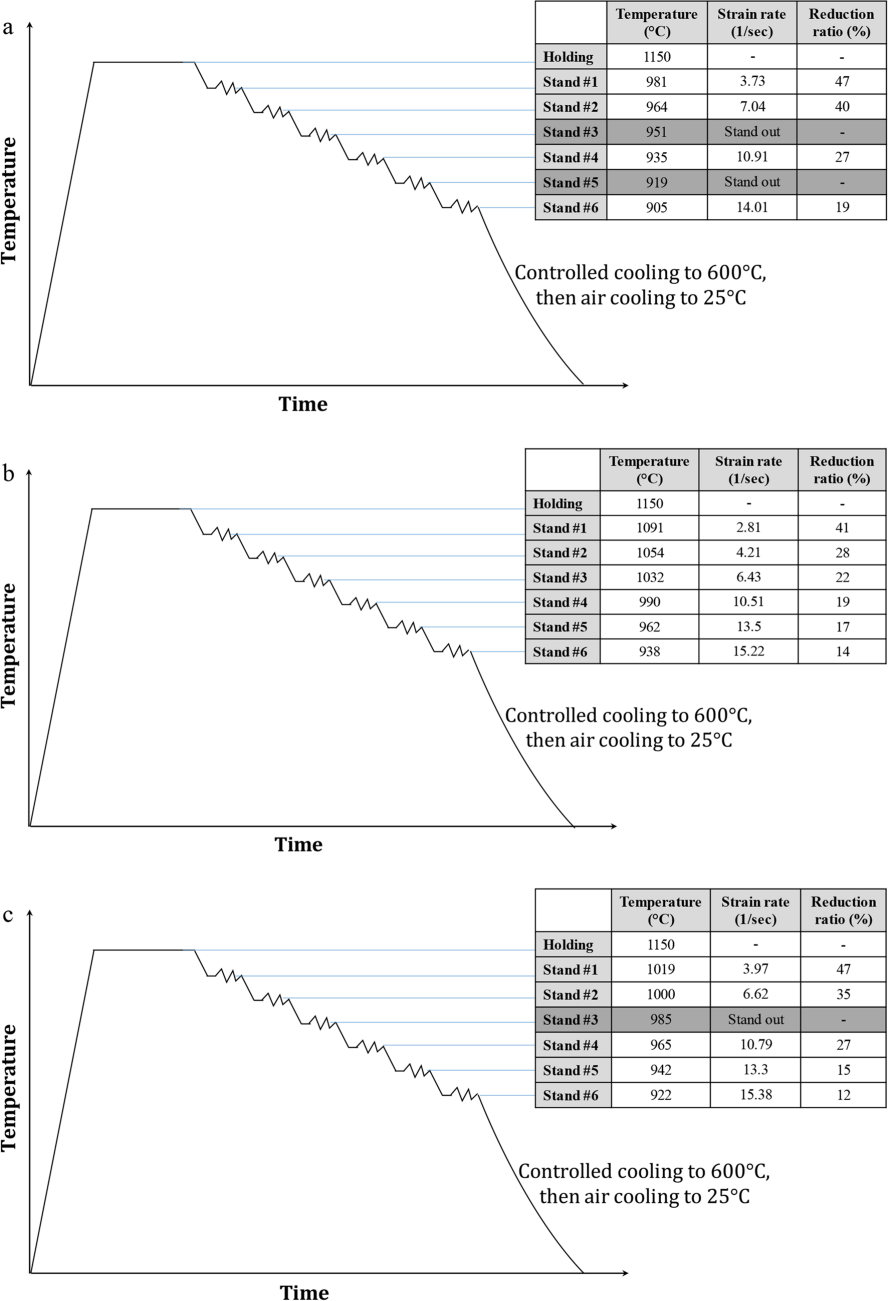
HSM deformation and cooling regimes at EZDK-HSM for the different studied alloys: (**a**) base, (**b**) 15Nb, and (**c**) 15Nb30B alloys.

In this study, three different low-carbon steel alloys with varying niobium and boron levels were made from iron ore that was directly reduced at the MIDREX direct reduction plant at Al Ezz Dekheila Steel Company (EZDK) in Alexandria, Egypt. Direct-reduced iron (DRI) was commercially called sponge iron, and it was charged with some additives into an electric arc furnace (EAF) to melt down, resulting in molten steel. After the molten steel was poured into a ladle, it was transported to the ladle furnace station (LF) for treatment. The treated molten steel was obtained by modifying its chemical composition. The chemical composition of the three studied steel alloys is presented in Table 1. The treated molten steel was turned into a compact strip production line (CSP) and cast in a thin slab continuous casting machine (TSC) into steel slabs of 52 mm thickness. The EZDK-CSP line scheme is shown in Fig. 1. Finally, the slabs in a hot strip mill (HSM) with six stands reach the final thickness of 10.0 mm, followed by an appropriate cooling regime on the run-out table, relatively slow cooling was applied for better formability before coiling. Figure 2 presents the HSM deformation and cooling regimes at EZDK-HSM for the different studied alloys.

**Table 1:** Chemical composition of the studied low carbon steel alloys.

Alloy	C (wt%)	Si (wt%)	Mn (wt%)	Nb (wt%)	Al (wt%)	B (ppm)	*N* (ppm)	Fe
Base	0.047	0.02	0.91	0	0.042	< 5	68	Balance
15Nb	0.050	0.04	0.88	0.015	0.032	< 5	46	Balance
15Nb30B	0.051	0.04	0.93	0.015	0.030	30	47	Balance

The chemical analysis was done by optical emission spectrometer (Model: Spectro-Ametec, Germany, Manufacturing year: 2013, Technique: Hyper-PMT + CCD, Standard: ASTM A751-14a (ASTM E415-15).

The T_nr_ was determined by obtaining the mean flow stresses (MFS) and deformation temperatures from Level II of EZDK-HSM (the mill log, which automatically records data during the rolling process). The flow stresses were calculated by using process setup calculation module software, mainly relying on force measurements of rolling stands with the corresponding strain. Online fixed pyrometers type “SYSTEM4-M1” with measuring range (600 ~ 1600 °C) and accuracy ± (0.004 × measured temperature) were used to measure temperatures. By plotting the flow stress at each stand of HSM versus the inverse of absolute deformation temperature and fitting the trend lines that represent the high and low deformation temperature regions, T_nr_ was determined as the intersection point of the two straight lines.

To investigate the effect of the different online production regimes for the studied alloys in the as-rolled condition on the mechanical properties, the quasi-static tensile test was carried out at room temperature according to “ISO 6892-1” using a Zwick/Roll (250 KN) tensile testing machine in the rolling direction.

The equilibrium phase evolutions and the thermodynamic calculations for the solubility of Nb/B in FCC austenite and BCC ferrite for the studied steel alloys were conducted using ThermoCalc software (TCFE 10 database). The prior austenite grain size of the as-rolled specimens was measured at elevated temperatures. The specimens were heated to 1200, 1150, 1100, 1050, 1000, 950, and 900 °C and then held for 20 min, followed by water quenching. After quenching, the specimens were prepared for metallography examination using a special etchant (5 gm FeCl_3_ + 5 drops HCl + 100 ml water) according to ASTM E 407.

**Figure 3:**
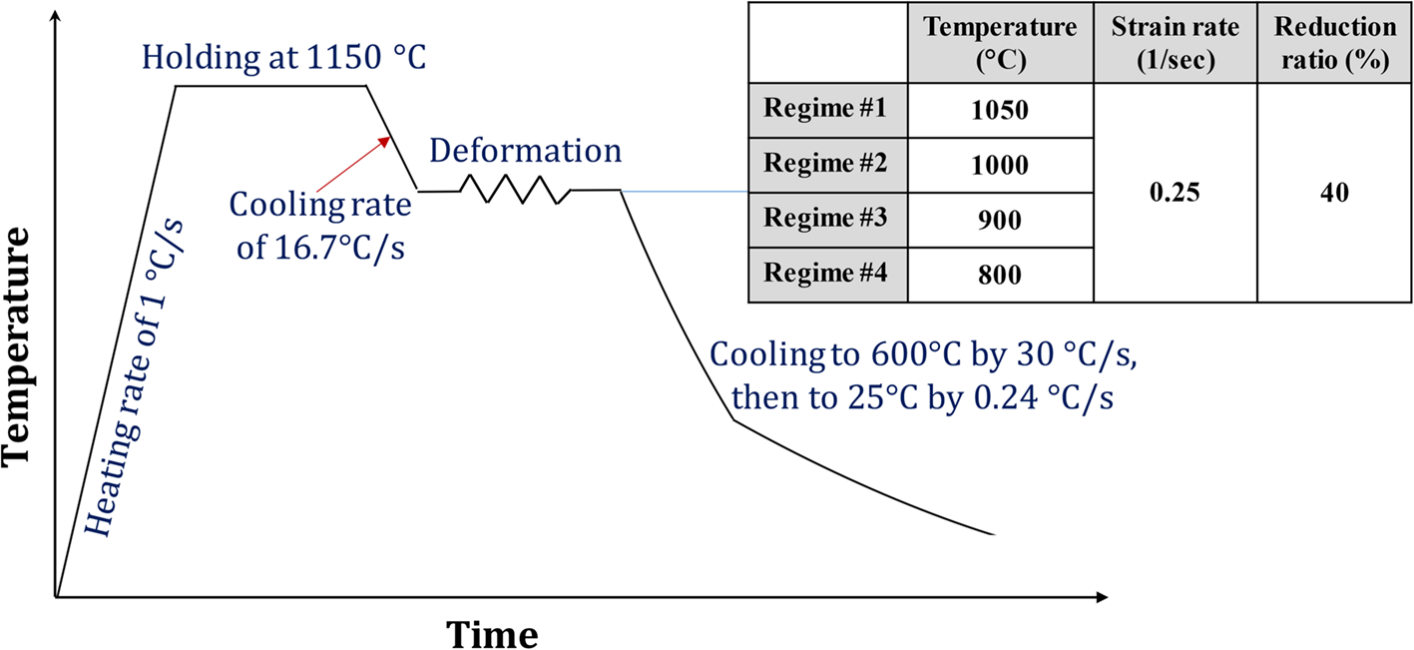
Single-pass TMCP regimes for Gleeble simulator.

Different TMCP regimes were applied to the as-rolled low-carbon steel alloys through a thermomechanical simulator (Gleeble 3500). The specimens for the thermomechanical simulator were prepared from the received strips as bars of 10 mm in diameter. Then each specimen was welded to the thermocouple using a thermocouple welding device. Finally, the specimen was fixed in the simulator test chamber. Single-pass TMCP regimes have been applied through hot compression tests in the rolling direction, keeping the total amount of deformation at 40%. The schematic of typical single-pass TMCP regimes is shown in Fig. 3. A relatively low heating rate of about 1.0 °C/s up to 1150 °C for all strategies has been undertaken to ensure a homogeneous structure before deformation and to simulate the industrial production procedure. After soaking specimens for 5 min at 1150 °C and controlled cooling to the deformation temperatures at a cooling rate of about 16.7 °C/s, about 40% reduction was applied in one pass at different deformation temperatures of 1050, 1000, 900, and 800 °C at a 0.25 s^−1^ strain rate. A high cooling rate of 30 °C/s to a temperature of 600 °C after deformation was applied in all TMCP regimes, followed by controlled cooling to room temperature by applying a low cooling rate of 0.24 °C/s.

The microstructure characteristics of the as-rolled and Gleeble specimens were examined on an Olympus optical microscope equipped with the digital camera model AXIOCAM MRC5 with an accuracy of ± 1.5%. The specimens were etched by nital reagent for 4–6 s to reveal the microstructure. Measuring the grain size was carried out at about one-third depth from the rolling surface or compression face for as-rolled and Gleeble specimens, respectively, by the plan-metric method according to ASTM E112-2013. Also, some selected specimens were investigated using a Type JEOL scanning electron microscope (SEM-JSM-IT200 Series) and energy dispersive spectroscopy (EDS).

## Results and discussion

### Thermodynamic calculations

**Figure 4:**
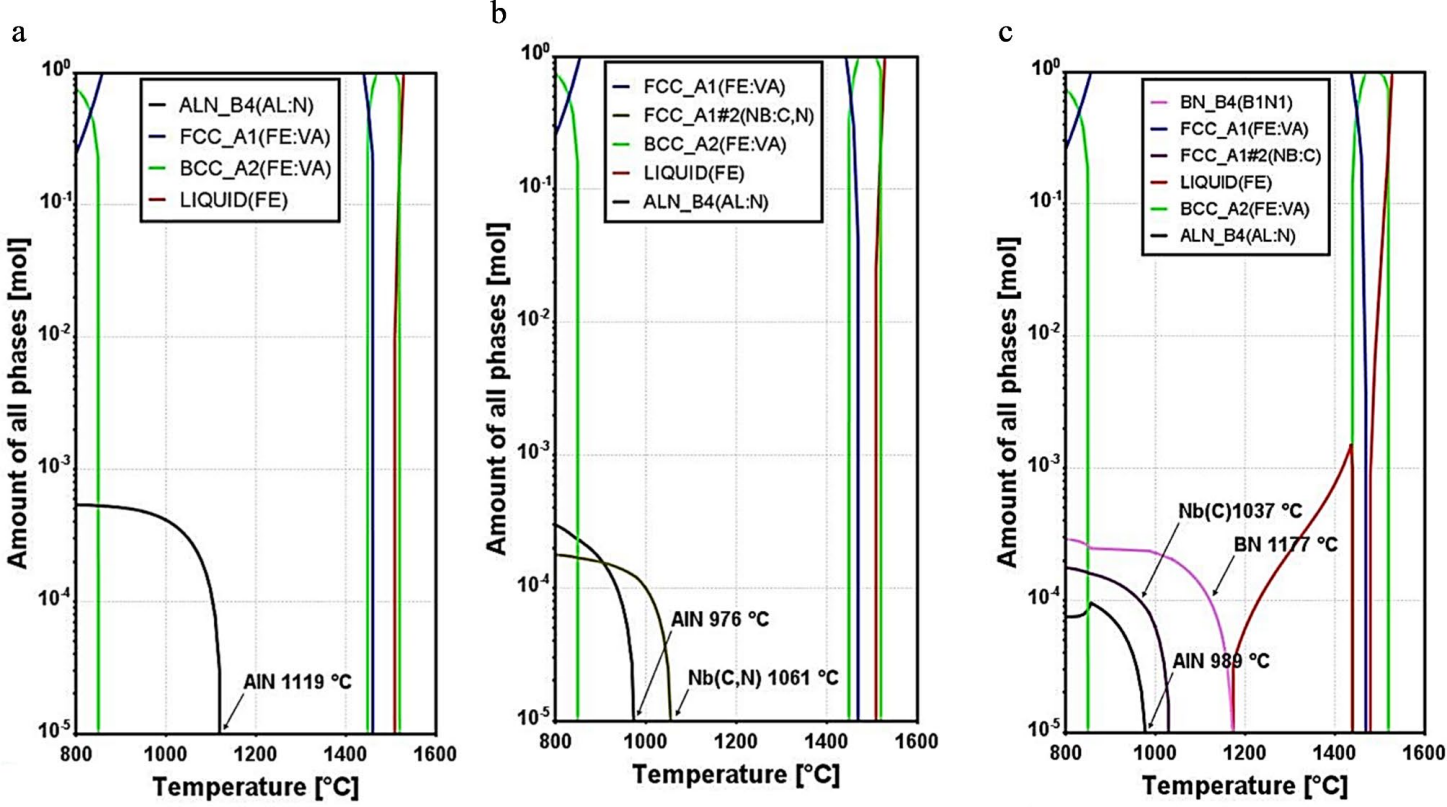
The equilibrium amount of all phases vs. temperature for the studied steel alloys was calculated using ThermoCalc software (TCFE 10 database): (**a**) base, (**b**) 15Nb, and (**c**) 15Nb30B alloys.

The equilibrium phase evolution with temperature of the investigated steel alloys is shown in Fig. 4. In the base alloy (Fig. 4a), all N goes to Al to form AlN at a high temperature of 1119 °C. In the 15Nb alloy, a definite amount of N goes to Nb to form Nb(C, N) at 1061 °C, which results in the formation of AlN at a lower temperature of 976 °C (Fig. 4b). For the 15Nb30B alloy, most of N goes to B to form BN at 1177 °C, thus neither NbN nor Nb(C, N) were formed, and only Nb(C) starts formation at a lower temperature of 1037 °C, then AlN starts formation at 989 °C (Fig. 4c). Calculations carried out by Pitakkorraras et al.^23^ using JMatPro® software for B-alloyed and B-free low-C-Al-killed steel showed that in the presence of B, BN precipitates start to form at a higher temperature (about 1210 °C) and AlN precipitates start to form at about 900 °C.

**Figure 5:**
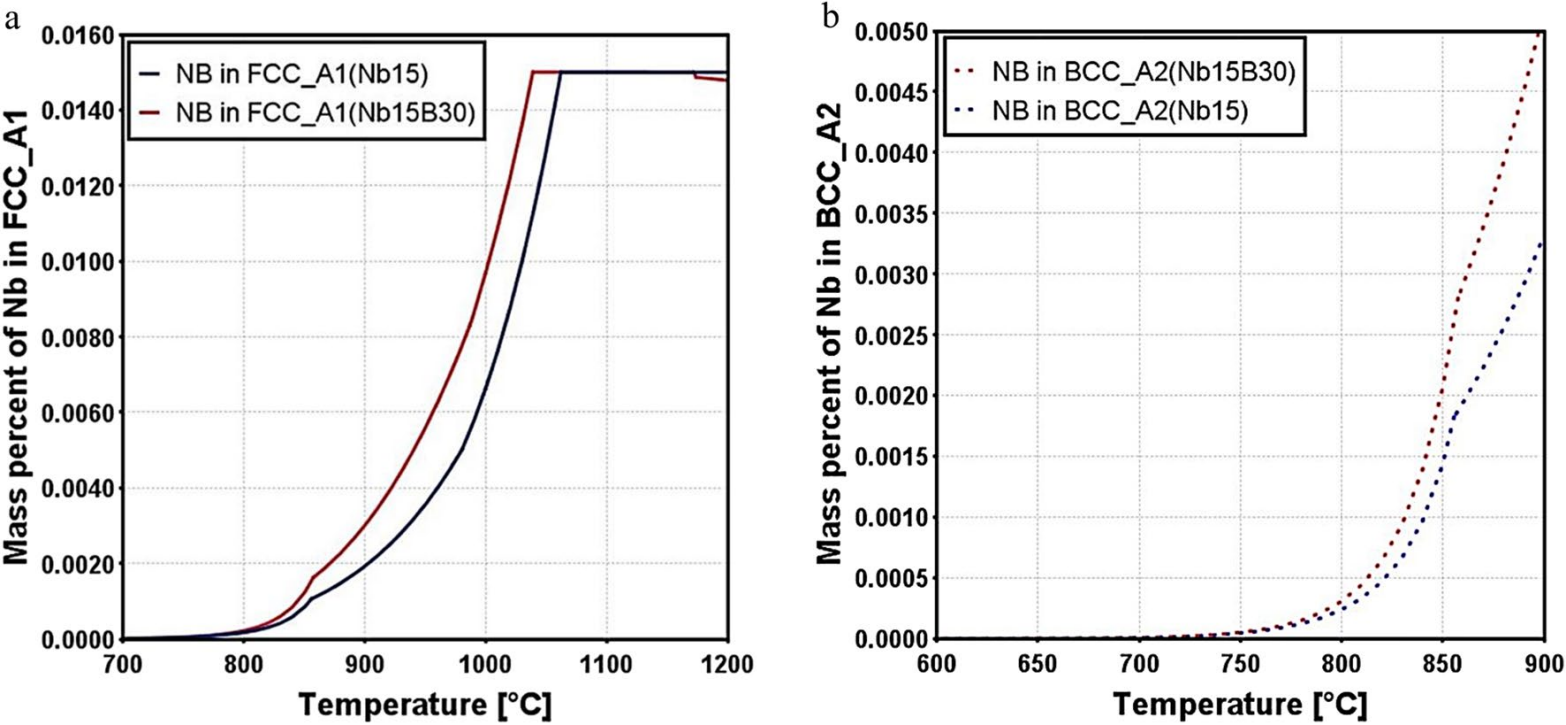
Solubility of Nb in FCC austenite (**a**) and BCC ferrite (**b**) for 15Nb and 15Nb30B alloys.

The thermodynamic calculation for the solubility of Nb in FCC austenite and BCC ferrite for 15Nb and 15Nb30B alloys is shown in Fig. 5. As the temperature increases, the solubility of Nb in both FCC austenite and BCC ferrite increases. The solubility of Nb in 15Nb30B alloy is higher than 15Nb alloy in both FCC austenite (Fig. 5a) and BCC ferrite (Fig. 5b) at all temperatures. This is attributed to the formation of BN at higher temperatures, which retards the precipitation of Nb-bearing precipitates. Below 800 °C, Nb has almost the same mass percentage in FCC austenite and BCC ferrite for both 15Nb and 15Nb30B alloys, respectively.

### Prior austenite grain size at high temperature

**Figure 6:**
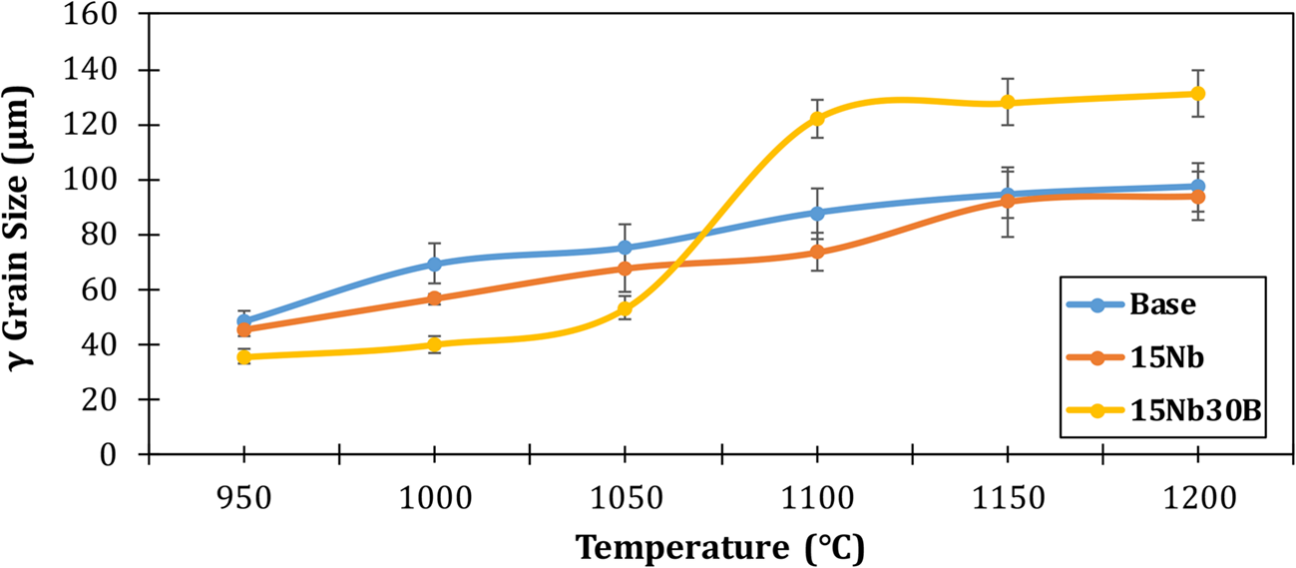
Effect of deformation temperature on prior austenite grain size.

Figure 6 shows the change in prior austenite grain size with a temperature in the range of 950–1200 °C for the studied steel alloys. Austenite grain size at 900 °C has been neglected due to the existence of two phases (ferrite and austenite phases). The lower the temperature, the lower the austenite grain size due to lower diffusion driving forces. 15Nb and base alloys reveal a uniform increase in the γ grain size, while the 15Nb30B alloy shows significant variation in the prior austenite grain size with temperature. γ shows much higher grain sizes at higher temperatures (≥ 1050 °C) that could be attributed to the formation of large BN precipitates that have a small effect on grain boundary pinning. However, at temperatures lower than 1050 °C, 15Nb30B alloy shows the lowest γ grain sizes than 15Nb and base alloys due to the formation of finer Nb(C) precipitates, as presented in the thermodynamic calculations (Fig. 4c), which has a strong effect on the grain boundary pinning and hence hinders grain growth.

### Evaluation of as-rolled alloys

#### As-rolled microstructure

**Figure 7:**
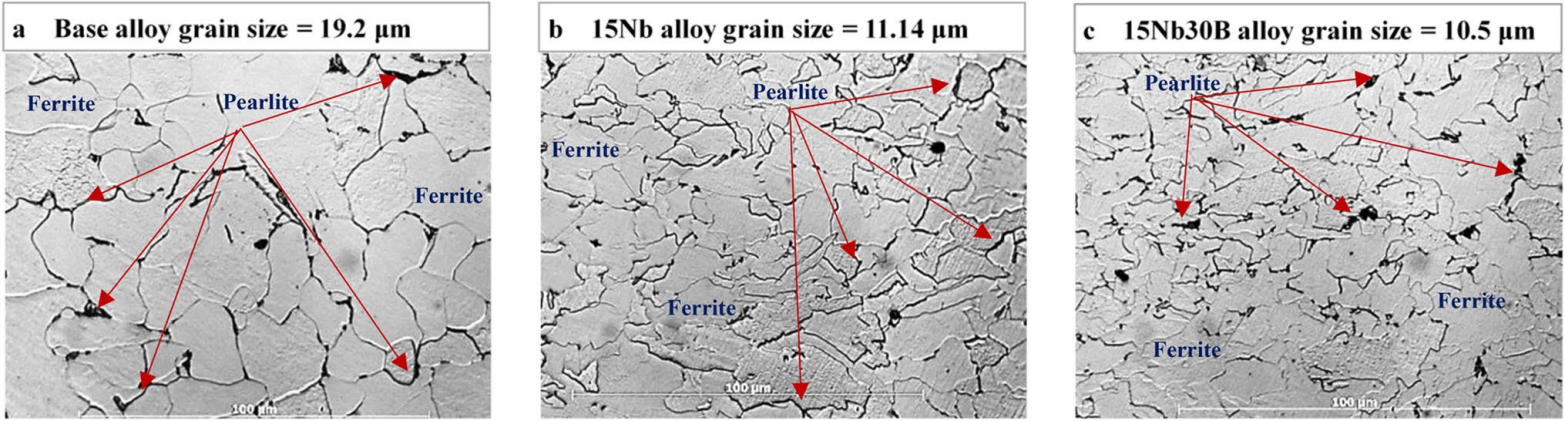
Microstructure of the as-rolled alloys: (**a**) base, (**b**) 15Nb, and (**c**) 15Nb30B alloys.

Figure 7 shows the microstructure of the base, 15Nb, and 15Nb30B as-rolled alloys. The microstructure of the three alloys shows a clear ferrite structure with small disparate pearlite grains due to low carbon content (~ 0.05%) and a slow cooling rate. The base alloy (Fig. 7a) showed the coarsest grain size of 19.2 μm. While 15Nb30B alloy (Fig. 7c) showed relatively finer and closer grain sizes of 10.5 μm in comparison with 15Nb alloy of 11.14 μm (Fig. 7b). On the other hand, no noticeable segregation was observed in the three alloys. Since all studied alloys were produced at HSM with the same slow cooling regime, differences in grain size for as-rolled alloys (Fig. 7) are only attributed to chemical composition and precipitation mechanisms.

#### Mechanical properties at room temperature

**Figure 8:**
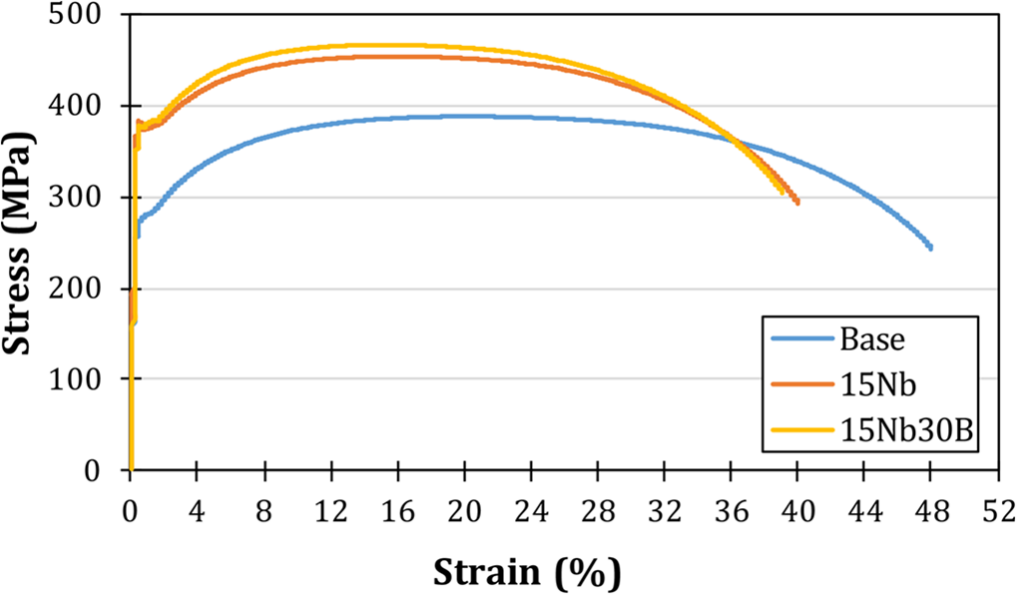
Tensile stress-strain curves for the as-rolled alloys.

**Figure 9:**
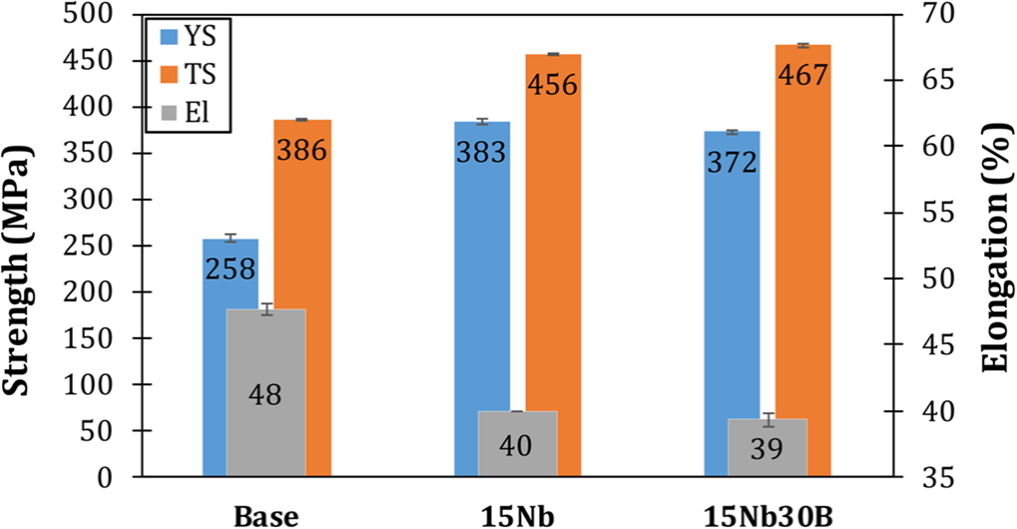
Tensile properties of the as-rolled alloys.

Figure 8 presents the tensile stress-strain curves of the as-rolled alloys carried out at room temperature; Fig. 9 illustrates the tensile properties of each alloy. The base alloy showed the lowest yield and ultimate tensile strength (YP and TS) and the highest elongation (El) values among the three alloys since the base alloy has the coarsest grain size and does not contain any strengthening elements. The 15Nb30B alloy shows relatively higher TS and lower El than the 15Nb alloy; this is attributed to a slightly finer structure. In their study, Hara et al.^24^ compared the effects of adding Nb and B alone and in combination. They found that adding Nb and B together can increase strength in low-C steel without sacrificing low-temperature toughness. This is due to the retardation of austenite to ferrite transformation as a result of an increase in segregated B along the austenite grain boundary before the austenite to ferrite transformation. B segregation occurs due to the suppression of the formation of Fe_23_(C, B)_6_ precipitates by the combined additions and the restriction of C diffusion towards the austenite grain boundary as a result of the precipitation of the fine dispersive Nb(C, N)^24^.

#### Determining of T_nr_

**Figure 10:**
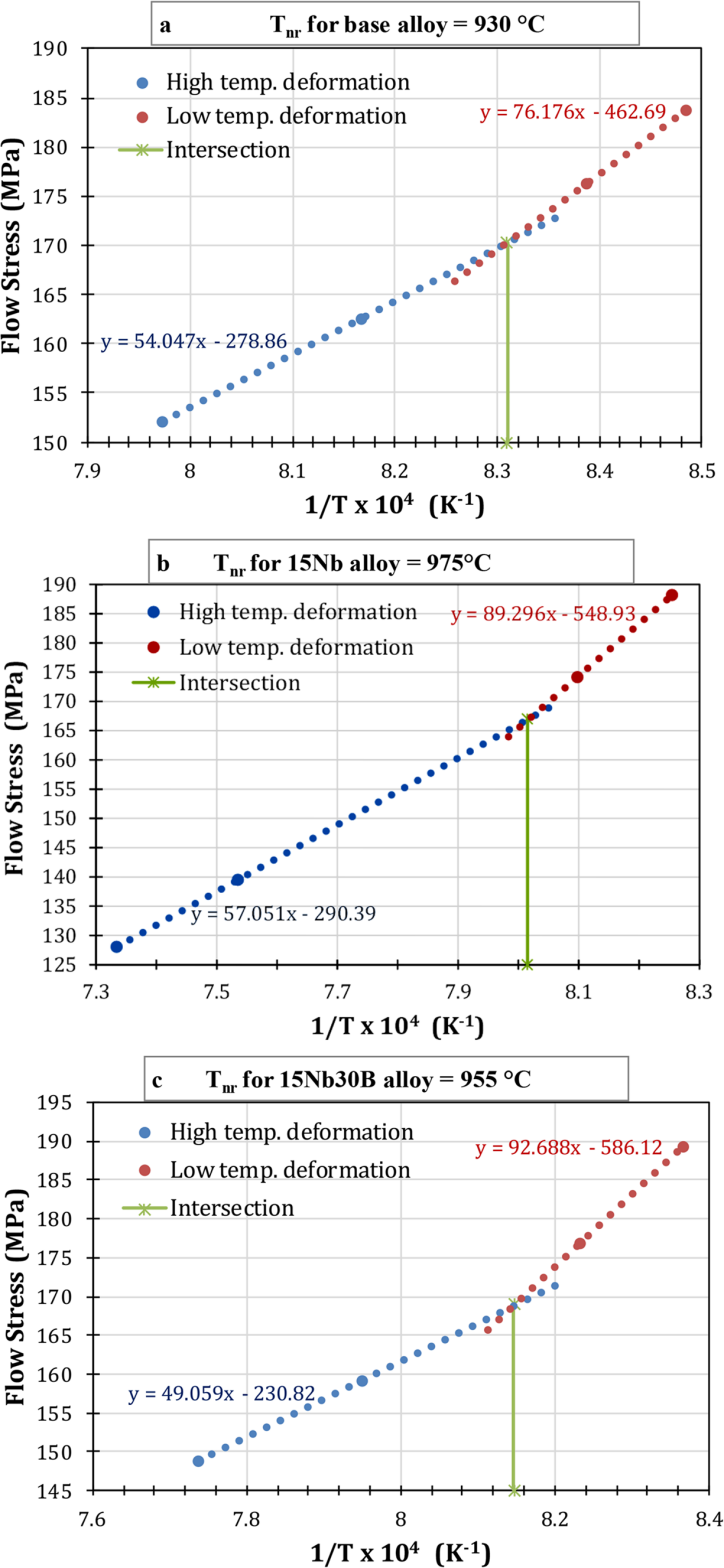
Mean flow stress during HSM rolling versus inverse absolute deformation temperature for: (**a**) base, (**b**) 15Nb, and (**c**) 15Nb30B alloys.

A crucial first step in designing controlled rolling regimes is the determination of the no-recrystallization temperature^2–4^. With a greater T_nr_, finish rolling can be done at higher temperatures, requiring lower rolling loads or applying larger reductions without consuming more energy^2,5^. To calculate T_nr_, successive rolling passes must be performed. For each pass, the mean flow stress (MFS) versus the inverse of the absolute deformation temperature has to be graphically represented^2,3,5^. Data from EZDK-HSM logs under continuous cooling conditions was used to calculate T_nr_ for the studied alloys. Figure 10 shows plots of the mean flow stress during rolling at each stand against 10,000/temperature in Kelvin for the studied alloys.

These MFS against 10,000/T plots can be partitioned into two stages: (i) a high-temperature deformation stage, a lower slope region that undergoes full recrystallization, in this stage the MFS slightly increases with decreasing temperature, and (ii) a low-temperature deformation stage, a higher slope region in which only partially or no recrystallization occurs, in this stage the MFS rapidly increases with decreasing temperature as the work-hardening is retained. A point of intersection is the T_nr_, which is produced by fitting straight lines between these two stages. Similar behavior was obtained by previous authors for Nb-bearing steels^2,3,8^. T_nr_ was found to be around 930 °C for the base alloy, 975 °C for the 15Nb alloy, and 955 °C for the 15Nb30B alloy. The addition of Nb to low-carbon steel causes T_nr_ to increase because NbC precipitates retard the recrystallization process. But when B is added to Nb-bearing steel, T_nr_ reduces because BN forms reducing NbC precipitate, which results in lower grain boundary pinning and less recrystallization retardation. Using an empirical formula, Dimatteo et al.^25^ calculated the T_nr_ for various HSLA steel compositions. They found the same outcomes when they assessed Nb and B’s single effects.

### Gleeble physical simulation

#### Gleebe TMCP flow behavior at high temperature

**Figure 11:**
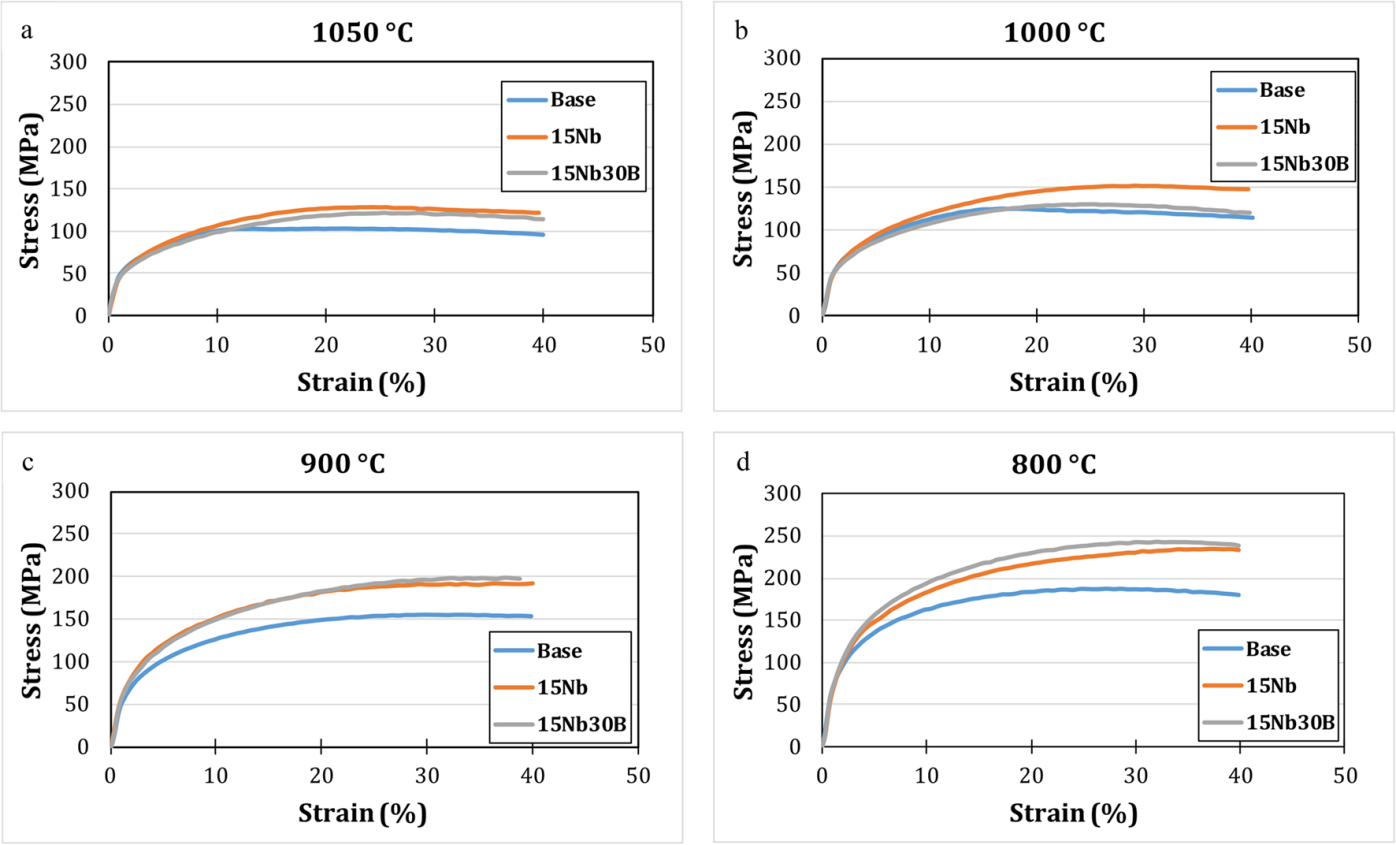
Flow behavior of the studied alloys at different deformation temperatures using Gleeble physical simulator: (**a**) 1050, (**b**) 1000, (**c**) 900, and (**d**) 800 °C.

Figure 11 shows stress-strain curves for the base, 15Nb, and 15Nb30B alloys of Gleeble compression tests carried out at 1050, 1000, 900, and 800 °C. The same strain of ~ 40% was applied for all alloys with a strain rate of 0.25 s^−1^. Flow stresses for 15Nb and 15Nb30B alloys are higher than the base alloy at all temperatures due to the presence of precipitates of NbN/Nb-carbonitride compounds and BN, according to thermodynamic calculations (Fig. 4).

Both 15Nb and 15Nb30B alloys almost have the same flow stress at 1050 °C (Fig. 11a). At this temperature, part of the Nb is soluble in the 15Nb alloys, as shown in the solubility diagram in Fig. 5a, and as Nb(C, N) starts to form at 1061 °C in the 15Nb alloy, a low amount of Nb(C, N) of about 1.5 × 10^−5^ mol. is formed at this temperature. While in the 15Nb30B alloy, most of the Nb is soluble in the structure, as shown in solubility diagram Fig. 5b, and no NbN is formed since most N goes to B to form BN of about 2 × 10^−5^ mol. (Fig. 5b), while Nb(C) starts to form at a lower temperature of 1037 °C, as illustrated in Figs. 4 and 5.

In comparison between 15Nb and 15Nb30B flow stresses at 1000 °C (Fig. 11b), 15Nb30B alloy shows lower strength since 15Nb alloy has a larger amount of Nb(C, N) at 1000 °C of about 10^−4^ mol., as shown in the phase evolution diagram Fig. 4b. While the 15Nb30B alloy (Fig. 4c) has no NbN at 1000 °C, it has only about 7 × 10^−5^ mol. of Nb(C), which has a lower strengthening effect in comparison with Nb(C, N).

15Nb30B alloy shows slightly higher flow stress than 15Nb alloy at 800 °C and 900 °C (Fig. 11c, d). This is attributed to strong solid solution strengthening due to the higher amounts of dissolving Nb in FCC austenite (Fig. 5a) and BCC ferrite (Fig. 5b) in the 15Nb30B alloy at these temperatures as compared to 15Nb alloy.

Taking T_nr_ into consideration, the base alloy shows lower strength at a temperature higher than T_nr_ (T_nr_ = 930 °C) due to softening that occurred due to recrystallization, which observably appeared in Gleeble stress-strain curves carried out at 1000 and 1050 °C. Similar behavior is observed for 15Nb (T_nr_ = 975 °C) and 15Nb30B (T_nr_ = 955 °C) alloys.

**Figure 12:**
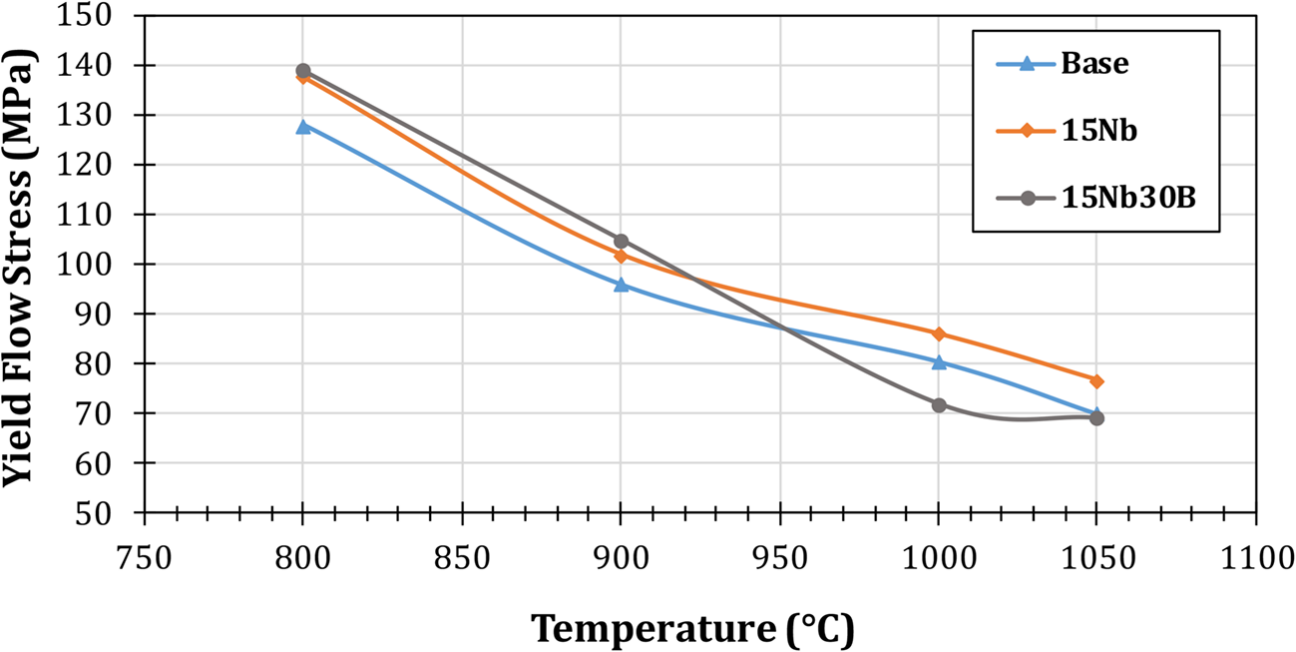
Relation between yield flow stress and deformation temperature for the studied steel alloys.

The relationship between yield flow stress and deformation temperature for the studied alloys is presented in Fig. 12. Both 15Nb and 15Nb30B alloys show adjacent flow behavior except at 1000 °C. This may be attributed to the formation of coarse BN precipitates in the 15Nb30B alloy and a small amount of NbC at this temperature, while the 15Nb alloy has a high amount of hard Nb(C, N) precipitates.

#### Microstructure of the Gleeble TMCP alloys

**Figure 13:**
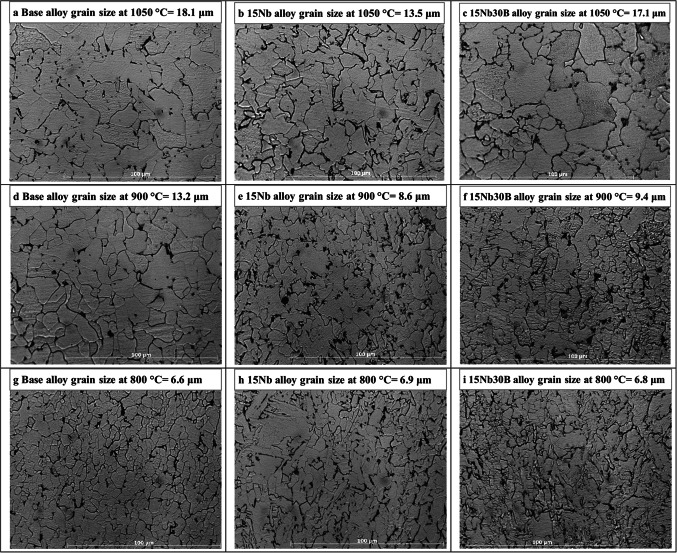
The microstructure of the studied alloys after Gleeble simulation tests.

Figure 13 shows the microstructure for the base, 15Nb, and 15Nb30B alloys after Gleeble hot compression simulation tests. Similar to as-rolled alloys, the microstructure of all alloys shows a clear ferrite structure with small disparate pearlite grains, which is attributed to the low C content. All alloys reveal coarse recrystallized grains formed during slow cooling after deformation at 1050 °C (Fig. 13a-c). The base alloy shows the coarsest grain size at deformation temperatures of 900–1050 °C since the base alloy has no content of Nb, which reacts as a grain refining element. The three alloys show almost similar grain sizes for Gleeble simulation tests carried out at 800 °C (Fig. 13g-i).

For tests carried out at 900 and 1050 °C, 15Nb alloy shows a finer grain size in comparison with 15Nb30B alloy due to the higher pinning effect of Nb(C, N). On the other hand, no noticeable segregation was observed in the three alloys.

**Figure 14:**
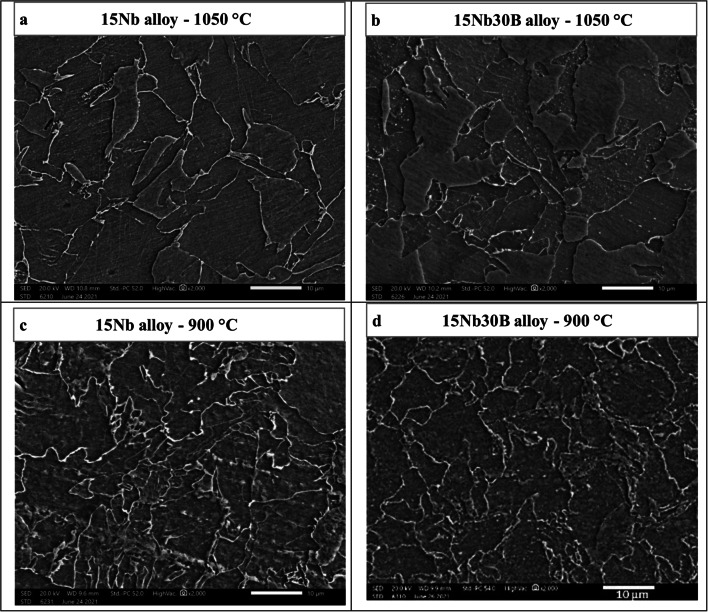
SEM micrographs for 15Nb and 15Nb30B alloys after Gleeble simulation tests at 1050 and 900 °C.

**Figure 15:**
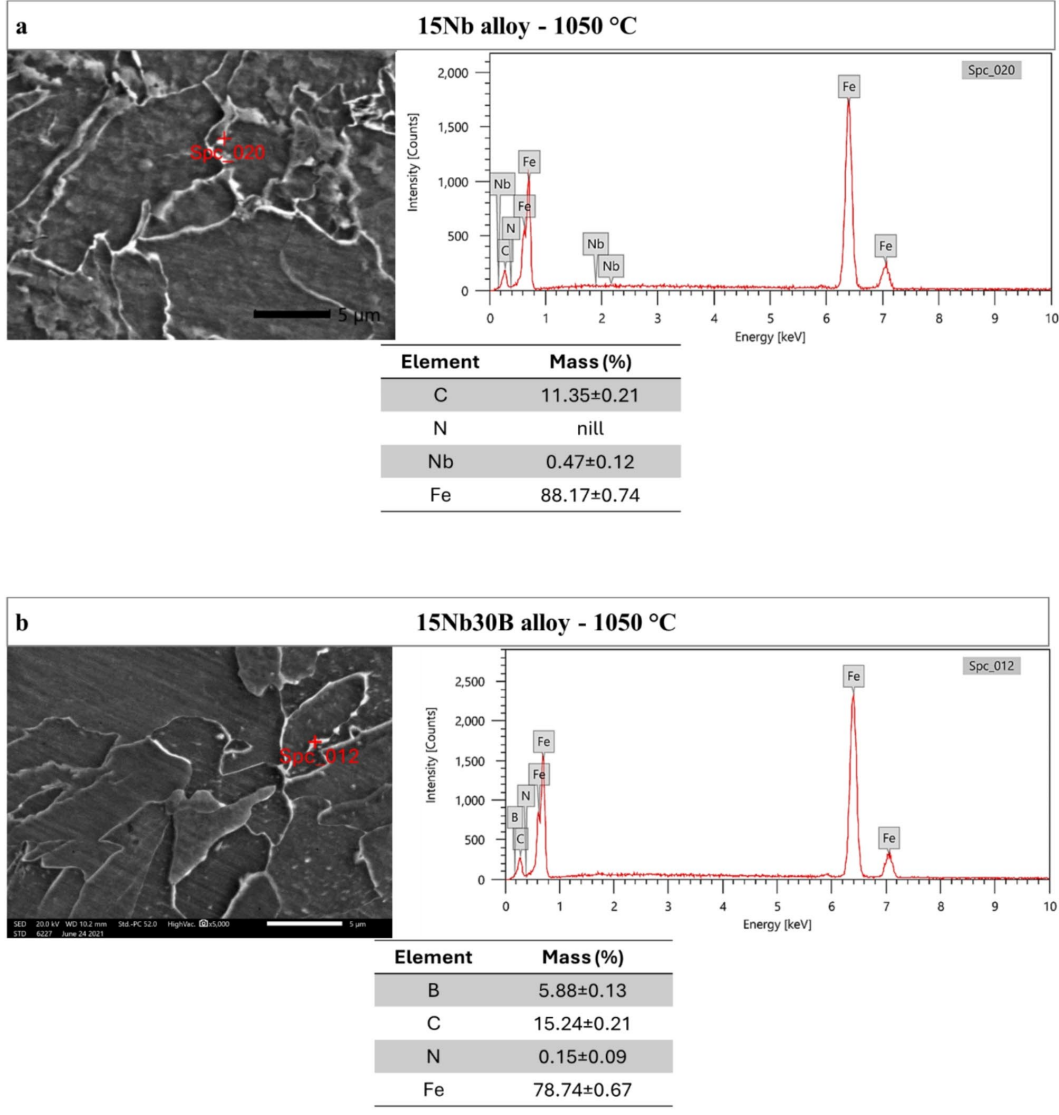
High magnification SEM micrographs and EDX analysis of the NbC and BN precipitates at the indicated red mark after Gleeble simulation tests at a deformation temperature of 1050 °C for: (**a**) the 15Nb and (**b**) 15Nb30B alloys, respectively.

Figure 14 shows the microstructure carried out by SEM for 15Nb and 15Nb30B alloys after Gleeble simulation tests carried out at 1050 and 900 °C. The 15Nb30B alloy shows bright precipitates, probably of BN. These precipitates are relatively coarser for a tested alloy at 1050 °C than the tested alloy at 900 °C due to the temperature and recrystallization effect that results in lower strength and softening at 1050 °C since coarser precipitates cause lower deformation resistance. The 15Nb alloy shows very small precipitates, probably of Nb(C, N). The higher magnification of the SEM micrograph with EDX analysis, shown in Fig. 15, reveals the presence of NbC and BN precipitates and their compositions. Previous work proved that precipitates in Nb-containing steel are much smaller in size than precipitates in B-Nb-containing steel for the same chemical composition, even with a small addition of B^24^. Yu and Kang^26^ also stated that since the precipitation of BN occurs at much higher temperatures, the BN precipitate size is larger.

## Conclusions

The current work investigates the mechanical properties, high-temperature flow behavior, and microstructural characteristics of Nb/B low-carbon steels produced by the CSP process. The addition of 30 ppm B to 0.015Nb-bearing low-carbon steel was found to have the following effects:

1. In the 15Nb30B alloy, the prior γ grain size exhibited two distinct behaviors at high temperatures. Grain boundary pinning is weakly affected by coarse BN precipitates, which result in larger grain sizes at temperatures over 1050 °C. Conversely, finer Nb(C) precipitates develop at temperatures below 1050 °C, leading to lower prior γ grain sizes.
2. The addition of B to the 15Nb alloy reduces T_nr_ from 975 to 955 °C, which requires forming at a much lower temperature in finishing passes. However, around 1177 °C, BN precipitates in the 15Nb30B alloy, vanishing Nb(N) and delaying Nb(C) precipitation, which results in a reduced yield flow stress at roughly 1000–1050 °C. Consequently, in comparison to 15Nb alloy, a lower rolling load is needed to deform 15Nb30B alloy at high temperatures.
3. Compared to the 15Nb alloy, the 15Nb30B alloy requires higher flow stress at a relatively low temperature (800 °C) to deform. At this low temperature, the solubility of Nb in both FCC austenite and BCC ferrite controls the deformation behavior.
4. The addition of B to Nb-bearing steel slightly refines the grain size of the as-rolled alloy, which in turn has little beneficial effect on the strength.

Finally, in terms of lowering the starting rolling load, reducing the finishing rolling temperature, and preserving the mechanical properties of the as-rolled strips produced by the CSP process, the research findings demonstrate the industrial value of adding B to Nb-bearing low C-steel.

## Data Availability

The data used to support the findings of this study are available from the corresponding authors upon request.
